# Identification of Key Genes for the Simultaneous Improvement of Fiber Strength and Lint Percentage in *Seg-D06-2* of Cotton by WGCNA

**DOI:** 10.3390/ijms27104180

**Published:** 2026-05-08

**Authors:** Shaoqi Li, Guangen Wang, Xi Zhang, Yi Liu, Sujun Zhang, Jianhong Zhang, Youlu Yuan, Junlan Li, Yuyuan Qian

**Affiliations:** 1 Key Laboratory of Cotton Biology and Genetic Breeding in Huanghuaihai Semiarid Area, Ministry of Agriculture and Rural Affairs, Hebei Key Laboratory of Cotton Bio-Breeding and Cultivation Physiology, National Cotton Improvement Center Hebei Branch, Institute of Cotton, Hebei Academy of Agriculture and Forestry Sciences, Shijiazhuang 050051, China; 2 National Key Laboratory of Cotton Bio-Breeding and Integrated Utilization, Anyang 455000, China

**Keywords:** cotton CSSLs, fiber strength, lint percentage, simultaneous improvement, RNA-seq

## Abstract

The simultaneous improvement of fiber strength (FS) and lint percentage (LP) is a critical objective for achieving high-quality and high-yield cotton production. Identifying key genes and their regulatory networks that govern the synergistic development of FS and LP is essential for achieving their simultaneous improvement. In our previous study, a stable chromosome segment, *Seg-D06-2*, was identified for its ability to concurrently enhance both FS and LP with high reliability. In the present study, homozygous individuals harboring the *Seg-D06-2* segment within a nearly uniform genetic background were selected to construct a large BC_6_F_2_ chromosome segment substitution line (CSSL) population comprising 3324 individuals. Extreme individuals characterized by simultaneous improvement in FS and LP, which shared similar genetic and phenotypic backgrounds, were subjected to comparative transcriptomic and weighted gene co-expression network analysis (WGCNA) at 0, 5, 10, 15, 20, and 25 days post-anthesis (DPA). The results highlighted the ’blue’ and ’yellow’ modules as being significantly associated with the simultaneous improvement of FS and LP. Four hub genes (*GH_D06G0542*, *GH_D06G1609*, *GH_D06G0627* and *GH_D06G2689*) and two DEGs (*GH_D06G0564* and *GH_D06G0723*) were identified in the ’blue’ module. Three hub genes (*GH_D06G0540*, *GH_D06G0558* and *GH_D06G0636*) and one DEG (*GH_D06G0527*) were identified in the ’yellow’ module. These 10 key genes likely play pivotal roles in regulating the synergistic development of FS and LP, warranting further investigation. The reliability of the RNA-seq data was confirmed by qRT-PCR. This study provides a valuable resource for molecular breeding aimed at the simultaneous improvement of FS and LP and offers new insights into the molecular mechanisms governing their synergistic development.

## 1. Introduction

Fiber strength (FS) and lint percentage (LP) are two pivotal indicators for evaluating cotton fiber quality and yield, respectively. A significant genetic negative correlation exists between these two traits, which has long been a bottleneck for the simultaneous improvement of both quality and yield [1,2,3]. The correlation coefficients between FS and LP typically fluctuate from −0.6 to −0.2; such wide-ranging fluctuations suggest that the interactive development of FS and LP is a process of dynamic coordination and trade-off [1,2,3,4]. This phenomenon provides the theoretical possibility of achieving simultaneous improvement of FS and LP at the level of fiber development.

The complete process of cotton fiber development comprises four distinct yet overlapping stages: initiation, elongation, secondary wall thickening (SWT), and maturation/dehydration [5,6]. During this process, at least three typical differences exist between high-FS *Gossypium barbadense* and high-LP *Gossypium hirsutum* which may be associated with the formation of fiber strength and lint percentage: (1) During the fiber initiation stage (−3 to 5 days post-anthesis, DPA), *G. hirsutum* ovules exhibit a higher density of fiber primordia cell protrusions, which favors an increase in LP. However, this also leads to competition for biomass accumulation among individual fiber cells, which is detrimental to the sustained development of high-quality fibers [6]. (2) During the rapid elongation stage (3–15 DPA), most key homologous genes in *G. barbadense* exhibit more active and persistent expression patterns [7,8], which promotes the formation of high-quality fibers and contributes to improved LP. (3) During the SWT stage (18–45 DPA), compared with *G. hirsutum*, the synthesis and deposition of cellulose in *G. barbadense* fibers follow a slower but more sustained developmental mode. This ensures substantial cellulose deposition around 30 DPA, a period characterized by the formation of large crystallites in the fiber crystalline regions. This enables the cellulose deposited throughout the boll period to be fully utilized within the fiber supramolecular structure, facilitating the formation of a high-crystallinity structure rich in compact large grains, thereby enhancing the mechanical properties of fiber bundles and forming high-strength cotton fibers [5,6,7,8,9,10]. These foundational theories of fiber development provide an effective reference for further screening key candidate genes and their interacting partners within localized genomic intervals for simultaneous improvement.

Based on the characteristics of fiber development, researchers have deciphered numerous genes involved in fiber development using molecular markers and high-throughput sequencing. For instance, MYB transcription factors such as *GhMYB109*, *GaMYB2*, *GhMYB25*, *GhMYB44*, and *GhCP* significantly regulate fiber initiation and elongation [5,11]. WRKY transcription factors *WRKY16* and *GhWRKY40* interact with *GhAP3* to prominently regulate fiber elongation [12,13]. The E3 ubiquitin ligase *GhXB38D* inhibits fiber elongation by ubiquitinating *GhACS4* and *GhACO1* [14], while *GhBZR3* suppresses elongation by inhibiting the expression of *GhKCS13* [15]. The transmembrane protein *GbTMEM209* negatively regulates fiber elongation through competitive interaction that inhibits the transcriptional activity of *GbHOX3* on downstream target genes such as *RDL1* and *EXPA1* [16]. Additionally, *GhBB2*, *GhCIPK10*, and *GhKRP6* have been proven to significantly influence fiber length [17,18]. *GhMYB102* and *GhMYB46* regulate the synthesis of the fiber secondary wall through a synergistic and competitive relationship by modulating genes such as *GhIRX10*, *GhCASPL1*, and *GhCAS4,7,8* [19,20]. *GhGASA24* significantly regulates fiber cell wall thickness [21]. ERF transcription factors (*GhERF5*, *7*, *9*, *12*, *10*) interact with the NAC transcription factor *GbNTL9* or the auxin response factor *GhARF7* to regulate fiber strength via the ethylene signaling pathway [22,23]. Furthermore, the endo-β-1,3-glucanase *GhPdBG* targets plasmodesmata, which serve as gates for intercellular channels, thereby regulating the genetic development of cotton fibers and trichome cells [24]. Another endo-β-1,3-glucanase, *GhGLU18*, promotes polysaccharide accumulation, cell wall thickening, cellulose synthesis, and tightens the fiber helical pitch, ultimately leading to increased fiber length and strength [25].

Weighted gene co-expression network analysis (WGCNA) has been widely applied as a powerful method to illustrate the genetic architecture underlying complex traits by extracting meaningful differences across the integration of large-scale transcripts and complex traits [26,27,28]. The gene sets and their co-expression modules, including hub genes which play a key role in the regulation of the gene expression network, can be identified effectively, as they are strongly and specifically associated with the target traits. WGCNA has been widely used to study fiber traits [27,29].

In our previous study, based on a BC_5_F_2_ segregating CSSL population derived from a high-FS *G. barbadense* cultivar (Hai1) and a high-LP *G. hirsutum* cultivar (CCRI45), we identified a chromosome segment, *Seg-D06-2*, which stably and simultaneously improved FS and LP across multiple environments. This segment explained a maximum phenotypic variation of 13.19% for FS and 7.80% for LP [30]. In the present study, homozygous individuals with a uniform genetic background harboring the target segment *Seg-D06-2* were screened from the BC_5_F_2_ segregating population using linked markers. These individuals were used as male parents and backcrossed with the recurrent parent CCRI45 to construct a large BC_6_F_2_ segregating population comprising 3324 individuals. Based on this constructed segregation population of 3324 BC_6_F_2_ individuals, extreme samples focusing on *Seg-D06-2* were selected. These samples, which shared similar genetic and phenotypic backgrounds but specifically differed in the simultaneous improvement (or lack thereof) of FS and LP, were subjected to comparative transcriptomic and WGCNA during fiber development. This study aims to utilize this novel research strategy to specifically mine key genes and regulatory networks associated with the synergistic development of FS and LP within the *Seg-D06-2* segment, providing essential resources for molecular breeding aimed at the simultaneous improvement of cotton fiber quality and yield.

## 2. Results

### 2.1. Genomic Characterization of the Candidate Region Seg-D06-2

**Figure 1:**
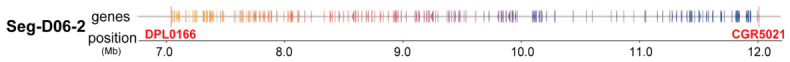
Gene structure of the candidate region *Seg-D06-2*.

*Seg-D06-2* was identified as a candidate chromosome segment for simultaneous improvement of cotton fiber strength and lint percentage in BC_5_F_2_ segregating CSSLs, which was tightly linked to the SSR markers DPL0166 and CGR5201 [30]. Based on the alignment of marker primer sequences against the TM-1 reference genome [31], the candidate genomic region of *Seg-D06-2* was delimited to D06: 7,389,785–12,082,570. The 4.69 Mb interval harbors a total of 203 annotated genes (*GH_D06G0523*–*GH_D06G0725*) (Figure 1).

### 2.2. Phenotyping and Extreme Sample Screening of the Segregating CSSLs

**Figure 2:**
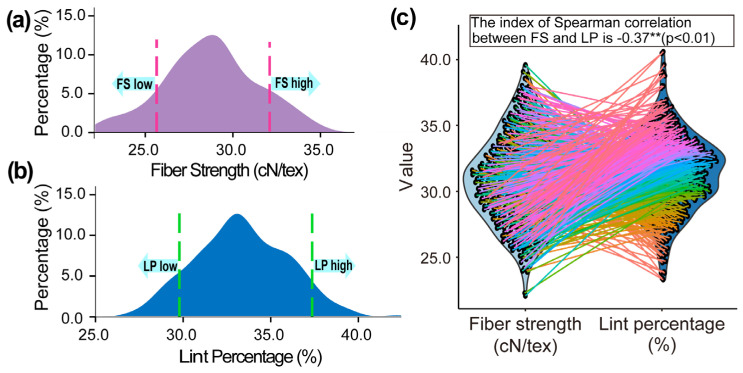
Phenotypes of fiber strength (FS) and lint percentage (LP) for BC_6_F_2_ segregating CSSLs. (**a**) Phenotypic distribution of FS of BC_6_F_2_ segregating CSSLs; (**b**) Phenotypic distribution of LP of BC_6_F_2_ segregating CSSLs; (**c**) correlations between FS and LP of BC_6_F_2_ segregating CSSLs.

Homozygous plants harboring the target segment *Seg-D06-2* with a uniform genetic background were identified based on the polymorphisms of the linked markers DPL0166 and CGR5201 in the BC_5_F_2_ segregating population. These selected individuals were then crossed as female parents with the recurrent parent CCRI45 and self-pollinated to generate a BC_6_F_2_ segregating CSSL population comprising 3324 individuals, which were then evaluated for FS and LP phenotypes. The phenotypic values of FS and LP displayed continuous, broad, and approximately normal distributions, with ranges of 22.43–36.96 cN/tex and 25.84–41.87%, respectively (Figure 2a,b). Notably, Spearman correlation analysis revealed a highly significant negative correlation between FS and LP within the population (r = −0.37, *p* < 0.01) (Figure 2c). The extensive crossing of the lines connecting individual trait values further visually demonstrates the thorough segregation and recombination of FS and LP within the segregating population (Figure 2c). Owing to this ideal phenotypic distribution, extreme individuals targeting Seg-D06-2, specifically those exhibiting either simultaneously high FS and high LP (HH), or low FS and low LP (LL), with similar genetic and phenotypic backgrounds, were accurately and sufficiently selected (Table 1).

In the five high FS and high LP (HH) individuals, the average performance of FS and LP was 34.05 cN/tex and 38.69%, respectively. In the five low FS and low LP (LL) individuals, the average performance of FS and LP was 24.85 cN/tex and 26.71%, respectively.

### 2.3. Evaluation and Analysis of Transcriptome

To explore the molecular basis underlying the simultaneous development of FS and LP regulated by genes within *Seg-D06-2*, we compared the transcriptomes of five extreme HH and five extreme LL individuals across six typical stages of fiber development (0, 5, 10, 15, 20, and 25 DPA). Equal amounts of RNA from three biological replicates were pooled to construct a single sequencing library. In total, 60 RNA-seq libraries were sequenced, and a total of 341.78 Gb clean data from 1367.13 million clean reads were generated. The clean data of each sample were more than 4.78 Gb, with a quality score Q30 > 89.62%, and the average GC content was 44.96%. The clean reads of each sample were mapped to the *G. hirsutum* reference genome of TM-1 [31], and the alignment rate ranged from 90.20% to 95.42%, with an average of 92.72% (Table S1).

**Figure 3:**
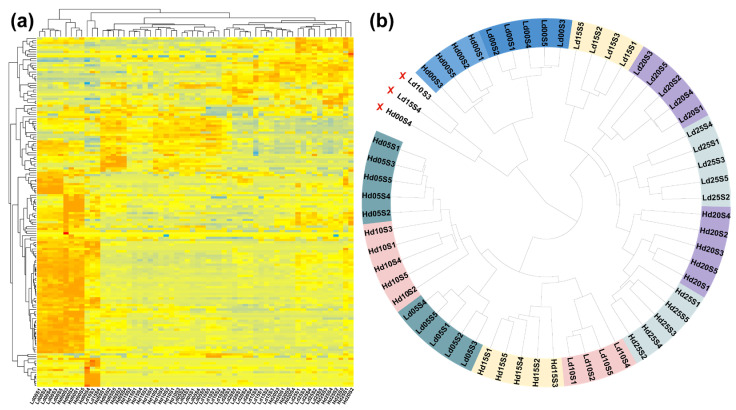
Evaluation of gene expression and sample homogeneity. (**a**) Heatmap of expressed genes in 60 samples; (**b**) clustering relationships among various sample types based on gene expression profiles, samples denoted by a red ‘×’ represent outliers that were omitted from downstream analyses.

Out of the 203 genes in *Seg-D06-2*, a total of 156 genes exhibited effective expression (counts ≥ 10.0) in at least one sample group (defined as all replicates representing a specific phenotype at a given stage). TPM values were utilized to assess the genome-wide normalized gene expression levels, after which the valid expression profiles of these 156 genes were extracted for downstream analysis (Figure 3a). To further validate the reliability of the samples and the sequencing data, a correlation analysis across all samples was conducted based on normalized TPM values. The results clearly demonstrated that, with the exception of Hd00S4, Ld10S3, and Ld15S4, samples representing different phenotypes and their respective developmental periods were significantly separated into distinct clusters (Figure 3b). To ensure data quality for downstream analyses, samples Hd00S4, Ld10S3, and Ld15S4 were excluded.

### 2.4. Identification of Differentially Expressed Genes Between Extreme Samples

**Figure 4:**
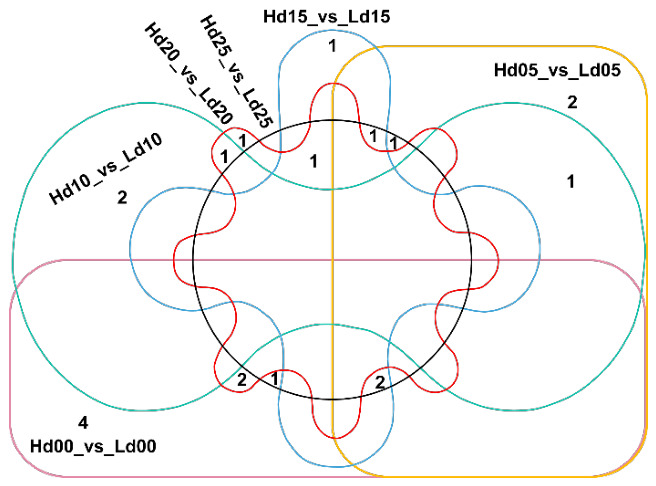
Statistics of DEGs across comparisons. Different comparison groups are distinguished by lines of various colors, and the numbers within represent the count of shared or unique genes between groups.

The differentially expressed genes (DEGs) between the HH and LL group were individually identified at each fiber developmental stage, revealing a total of 36 significant differential expression relationships involving 20 genes (Figure 4). Specifically, at 0 DPA, in the HH group relative to the LL group (Hd00_vs_Ld00), three significantly up-regulated genes (*GH_D06G0555*, *GH_D06G0601*, and *GH_D06G0680*) and six significantly down-regulated genes (*GH_D06G0523*, *GH_D06G0526*, *GH_D06G0564*, *GH_D06G0569*, *GH_D06G0570*, and *GH_D06G0723*) were identified. At 5 DPA, six significantly up-regulated genes (*GH_D06G0568*, *GH_D06G0569*, *GH_D06G0570*, *GH_D06G0586*, *GH_D06G0613*, and *GH_D06G0717*) and one significantly down-regulated gene (*GH_D06G0608*) were identified in the Hd05_vs_Ld05 comparison. At 10 DPA, six significantly up-regulated genes (*GH_D06G0620*, *GH_D06G0632* and *GH_D06G0717*) and one significantly down-regulated gene (*GH_D06G0533*) were identified in the Hd10_vs_Ld10 comparison. At 15 DPA, three significantly up-regulated genes (*GH_D06G0568*, *GH_D06G0569* and *GH_D06G0570*) and two significantly down-regulated genes (*GH_D06G0527* and *GH_D06G0536*) were identified in the Hd15_vs_Ld15 comparison. At 20 DPA, two significantly up-regulated genes (*GH_D06G0531*, and *GH_D06G0620*) and three significantly down-regulated genes (*GH_D06G0523*, *GH_D06G0526*, and *GH_D06G0527*) were identified in the Hd20_vs_Ld20 comparison. At 25 DPA, three significantly up-regulated genes (*GH_D06G0568*, *GH_D06G0569*, and *GH_D06G0570*) and three significantly down-regulated genes (*GH_D06G0527*, *GH_D06G0564* and *GH_D06G0608*) were identified in the Hd25_vs_Ld25 comparison.

The fold changes of DEGs ranged from 2.01 to 6.04 (*p* < 0.05). Among these, 10 DEGs were identified as significantly differentially expressed in only one of the six developmental stages, while six, two, and two genes were differentially expressed in two, three, and four stages, respectively (Figure 4). Interestingly, with the exception of *GH_D06G0569* and *GH_D06G0570*, which were differentially expressed across four stages and exhibited down-regulation in Hd05_vs_Ld05 but up-regulation in the other comparison groups (Hd05_vs_Ld05, Hd05_vs_Ld05 and Hd05_vs_Ld05), the expression trends of other genes identified in two or three stages remained consistent across different developmental periods.

### 2.5. WGCNA of Actively Expressed Genes

**Figure 5:**
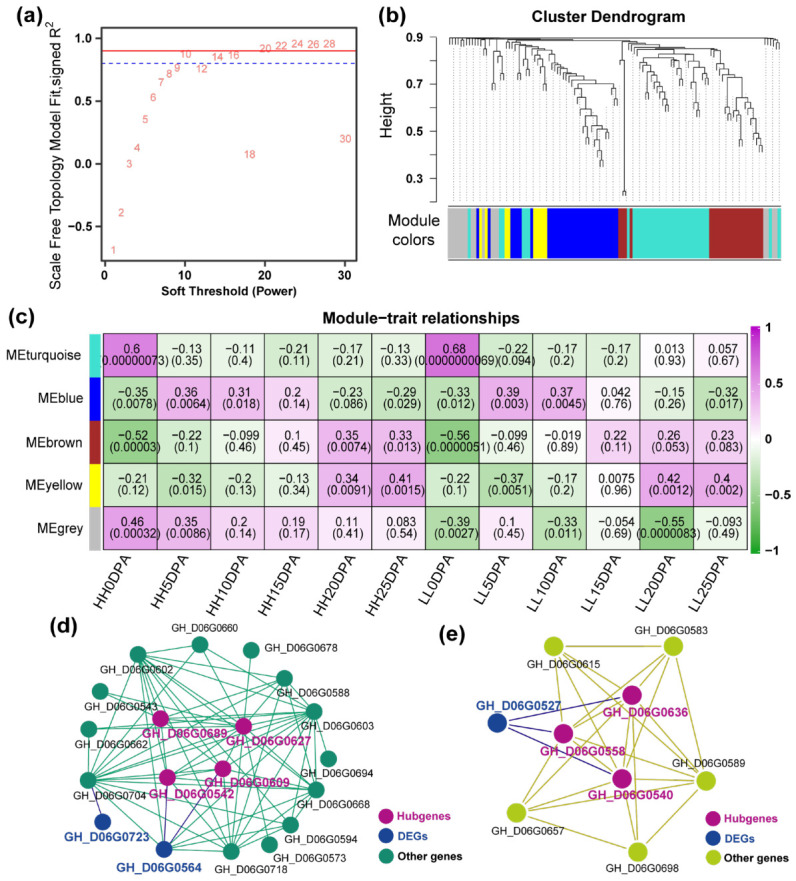
Analysis of co-expression network. (**a**) Soft threshold of scale-free network in WGCNA, the corresponding power values were marked with red numbers; (**b**) cutting clustering tree of gene co-expression network, the gene co-expression modules were represented by different color bands below the dendrogram; (**c**) correlation between gene co-expression network module and traits; (**d**) co-expression network of genes in blue module; (**e**) co-expression network of genes in yellow module.

In order to identify the specific gene sets that are strongly correlated with the simultaneous improvement of fiber strength and lint percentage, co-expression modules were generated by WGCNA using the actively expressed genes of 57 samples. The power of β = 10 (R^2^ = 0.93) was selected as a soft threshold to ensure a scale-free network (Figure 5a). Some genes with a higher correlation coefficient were clustered into the same cluster, and then the dynamic cutting method was used to cut the branches into 10 different modules. The modules with similar expression patterns were re-merged according to a correlation coefficient greater than 0.8. As a result, a total of five distinct modules associated with the specific expression profiles of different samples was obtained (Figure 5b,c). The turquoise module was the largest, comprising 36 genes, whereas the yellow module was the smallest, consisting of only nine genes. The grey module represents genes which cannot be classified into any one module and/or whose TPM < 1 in more than 50% of the samples (Figure 5b,c).

The correlation coefficients ranged from −0.56 to 0.68 between trait features of each sample and module eigengenes (Figure 5c). The module eigengene of ‘turquoise’, harboring 36 genes, was highly significantly positively associated with both HH and LL trait features at 0 DPA. The module eigengene of ‘blue’, harboring 32 genes, was significantly positively associated with both HH and LL trait features at 5 and 10 DPA, but significantly negatively associated with them at 0 and 25 DPA. The module eigengene of ‘brown’, harboring 23 genes, was significantly positively associated with both HH and LL trait features at 20 and 25 DPA, but highly significantly negatively associated with them at 0 DPA. The module eigengene of ‘yellow’, harboring nine genes, was significantly associated with both HH and LL trait features at 10, 20 and 25 DPA. No module is significantly associated with the trait features for 15 DPA.

### 2.6. Identification of Co-Expression Networks and Hub Genes of Candidate Modules

The eigengenes of both the ‘blue’ and ‘yellow’ modules were significantly associated with HH and LL trait features during the initial elongation (0, 5 and 10 DPA) and secondary wall rapid thickening stages (25 DPA) of fiber development (Figure 5c). We believe that genes significantly associated with both developmental stages merit special attention regarding their roles in regulating the coordinated development of fiber strength and lint percentage. Therefore, these two modules were designated as primary candidate modules, and their internal co-expression networks were deciphered (Figure 5d,e). There were four and three hub genes (purple) identified from ‘blue’ and ‘yellow’ modules, respectively, basing on the criteria of KME ≥ 0.95 and gene significance ≥ 0.50 in each group (Figure 5d,e). Intriguingly, although the hub genes in both modules were not identified as DEGs, they might play a role in regulating the expression of DEGs.

### 2.7. Functional Annotations of Key Genes

**Figure 6:**
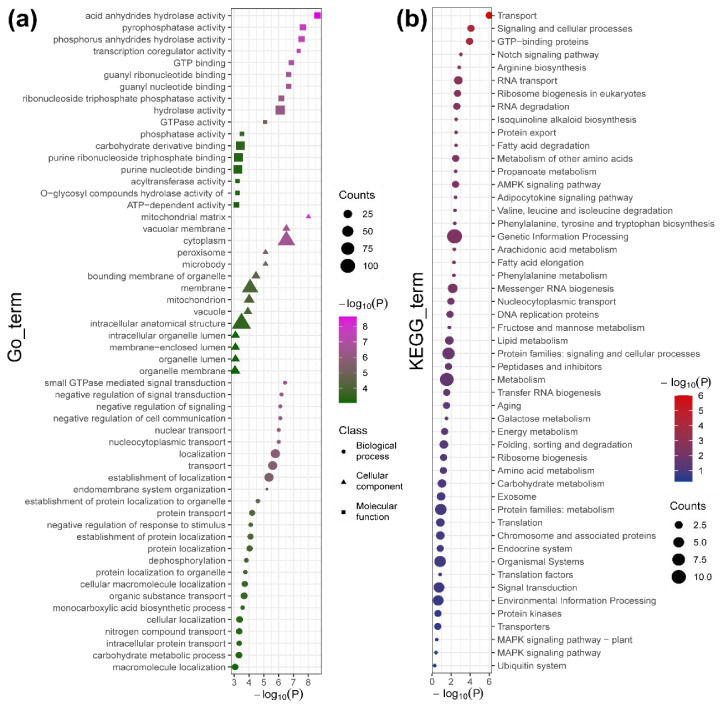
Functional annotation of actively expressed genes. (**a**) GO enrichment; (**b**) KEGG enrichment.

To identify and confirm the roles of the candidate modules in the formation of FS and LP, the actively expressed genes were annotated using functional annotation, GO enrichment, and KEGG pathway enrichment (Figure 6a,b).

The results of the functional annotation showed that 147 genes were accurately annotated based on the high homology with *Arabidopsis thaliana* (Table S2). The results of GO enrichment showed that 136 DEGs were highly significantly enriched in 10, 9, and 10 terms of biological processes, cellular components, and molecular functions, respectively (Figure 6a). In the biological process category, the unigenes were prominently enriched in small GTPase-mediated signal transduction (GO:0007264), negative regulation of cell communication (GO:0010648), nucleocytoplasmic transport (GO:0006913), localization (GO:0051179), transport (GO:0006810), establishment of protein localization to organelle (GO:0072594) and protein transport (GO:0015031). In the cellular component category, the unigenes were significantly enriched in the mitochondrial matrix (GO:0005759), vacuolar membrane (GO:0005774), cytoplasm (GO:0005737) and bounding membrane of organelle (GO:0098588). In the molecular function category, the unigenes were prominently enriched in hydrolase activity, acting on acid anhydrides (GO:0016817), pyrophosphatase activity (GO:0016462) and GTP binding (GO:0005525) (Figure 6a). In the KEGG pathways analysis, the unigenes were significantly enriched into 21 pathways, which were prominently related to ‘Transport’, ‘signaling and cellular processes’, ‘GTP-binding proteins’, ‘RNA transport’, ‘Ribosome biogenesis in eukaryotes’, ‘RNA degradation’, ‘Protein export’, ‘Genetic Information Processing’, and so on (Figure 6b).

Of particular note, four hub genes within the “blue” module, *GH_D06G0542*, *GH_D06G1609*, *GH_D06G0627*, and *GH_D06G2689*, were significantly enriched in terms related to material transport and energy metabolism on membrane structures within cells. Their specific functional roles are as follows: *GH_D06G0542* functions as a calmodulin-binding receptor-like cytoplasmic kinase (CRCK) gene, serving as a downstream effector in calcium signaling pathways; *GH_D06G0609* is a 3-ketoacyl-CoA synthase (KCS) gene that regulates the biosynthesis of very-long-chain fatty acids (VLCFAs), thereby influencing fiber cell elongation and cell wall formation; *GH_D06G0627* encodes an α/β-hydrolase involved in membrane lipid synthesis and remodeling, as well as the modification and degradation of cell wall polysaccharides; *GH_D06G0689* is an RNA-binding CRM domain protein gene, which is essential for maintaining cellular energy metabolism and regulating organellar RNA maturation. Three hub genes of the “yellow” module, *GH_D06G0540*, *GH_D06G0558* and *GH_D06G0636*, were significantly enriched in terms related to macromolecule localization and energy metabolism across membrane structures within cells. Their specific functional roles are as follows: *GH_D06G0540* is a calcium-dependent lipid-binding (CaLB domain) family protein; *GH_D06G0558* encodes an indole-3-acetic acid inducible 11; *GH_D06G0636* is a serine-rich protein-like protein.

### 2.8. Validation of Expression Profiles of Key Genes by qRT-PCR

**Figure 7:**
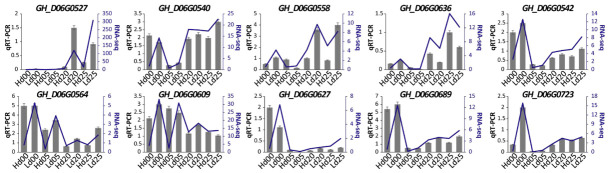
Validation of expression profiles by qRT-PCR.

To verify the reliability of the RNA-seq data, 10 representative genes were selected for qRT-PCR validation at 0, 5, 20, 25 DPA. These included six genes from the “blue” module (four hub genes and two DEGs) and four genes from the “yellow” module (three hub genes and one DEG) (Figure 5d,e). As expected, most of the selected genes showed a similar trend of expression profiles between qRT-PCR and RNA-seq across samples, particularly the expression DEGs (Figure 7), which confirmed the reliability of the RNA-seq data in this study.

## 3. Discussion

Extensive research has been conducted to identify genome-wide effect loci and their genetic mechanisms underlying the development of cotton fiber strength (FS) and lint percentage (LP), providing essential genetic resources for their improvement [2,3,5,7,9,17,19,20,21,22,23]. However, studies specifically focusing on their simultaneous improvement, particularly those investigating the regulatory networks of specific genetic loci, remain scarce. The present study focused on a previously identified pivotal chromosome segment, *Seg-D06-2*, which can concurrently enhance both FS and LP. Its significant effects have been directly or indirectly verified in multiple previous reports [3,32,33]. Utilizing chromosome segment substitution lines (CSSLs) with a relatively simple genetic background, we reconstructed a large BC_6_F_2_ segregating population comprising over 3000 individuals through backcrossing and self-pollination. Through extensive efforts, fiber samples from all individuals across six typical developmental stages were collected and preserved. Following fiber harvesting and phenotypic data collection, five extreme individuals demonstrating simultaneous improvement in FS and LP, along with five individuals lacking such simultaneous improvement, both of which shared nearly identical backgrounds for other traits, were reliably selected. RNA was extracted from their fibers at various stages for RNA-seq and expression regulatory network analysis. This strategy and approach represent a bold and innovative attempt in this research field.

The phenotypic values of FS and LP in BC_6_F_2_ segregating CSSLs displayed continuous, broad, and approximately normal distributions, with thorough segregation and recombination of FS and LP (Figure 2), which provided an ideal material basis for subsequent genetic analysis.

The WGCNA helped us to focus on the ‘blue’ and ‘yellow’ modules, in which eigengenes were significantly associated with HH and LL trait features during the initial elongation (0, 5 and 10 DPA) and secondary wall rapid thickening stages (25 DPA) of fiber development (Figure 5c). This concurrent association is a critical feature regulating the simultaneous development of fiber strength and lint percentage [5,6,34]. Thus, these two modules were identified as key candidate modules.

Weighted gene co-expression network analysis (WGCNA) revealed that no modules were significantly correlated with the trait features at 15 DPA. However, multiple modules were significantly associated with trait features at 0 DPA and 25 DPA. This indicates that the crucial functional genes within the *Seg-D06-2* interval predominantly regulate the synergistic development of FS and LP during the 0 DPA and 25 DPA stages, which is consistent with the initiation of fiber development at 0 DPA and the rapid thickening of the secondary cell wall at 25 DPA. At 0 DPA, the spatial organization of cellular components in long fiber cells is established, forming favorable cellular structures for rapid elongation, including the formation of a large central vacuole and the extensive attachment of ribosomes to the endoplasmic reticulum. Cell wall relaxation and vacuolar turgor are the internal driving forces for fiber cell expansion, while the quantity and state of ribosomes determine the extent and thickness of fiber elongation [5,6,34,35,36,37]. In this study, hub genes identified in two key modules were highly involved in functional networks related to cellular structure establishment and the precise localization of organelles, cell membranes, and vacuoles. At 25 DPA, during the rapid synthesis and deposition of secondary wall cellulose, cellulose microfibrils crystallize through covalent cross-linking, accompanied by the formation of the fiber supramolecular structure [5,35,36,38]. Key genes within the modules were related to signal transduction, substance localization and transport, and energy metabolism. The coordinated expression of these genes may contribute to the rhythmic arrangement of cellulose macromolecules in the crystalline regions of raw cotton fibers, leading to high orientation and facilitating the formation of dense, large crystallites in conjunction with cellulose deposition [5,29,35,36,38], thereby realizing the synergistic development of FS and LP.

Interestingly, none of the hub genes in the two key candidate modules were differentially expressed genes (DEGs). In the ‘blue’ module, two DEGs (*GH_D06G0564* and *GH_D06G0723*) were detected. Among them, *GH_D06G0564* formed direct co-expression regulatory relationships with the hub genes *GH_D06G0542* and *GH_D06G0609*, while *GH_D06G0723* formed indirect co-expression regulatory networks with numerous genes in the module, including four hub genes, via *GH_D06G0704* (Figure 5d). In the ‘yellow’ module, one DEG (*GH_D06G0527*) was detected, which formed direct co-expression regulatory relationships with three hub genes (*GH_D06G0540*, *GH_D06G0558*, and *GH_D06G0636*) (Figure 5e). The phenomenon of significant co-expression between DEGs and non-DEGs may be attributed to the relatively strict threshold (log2FC > 2) used during DEG analysis, which might have led to the exclusion of some genes that actually exhibited expression differences. For instance, validation via qRT-PCR and RNA-seq demonstrated that the hub gene *GH_D06G0636* exhibited differential expression at 20 and 25 DPA, while the hub gene *GH_D06G0627* showed differential expression at 0 DPA. Traditional DEG screening may inadvertently filter out critical upstream regulators that maintain relatively stable but biologically vital basal expression levels. Therefore, the integration of WGCNA provides a complementary and powerful approach to capturing these ‘hidden’ hub regulators, demonstrating that genes driving phenotypic variations are not strictly limited to those exhibiting large-scale differential expression. Furthermore, the majority of DEGs were positioned downstream in the regulatory network. This hierarchical architecture may reflect the inherent regulatory complexity of biological systems. Hub genes often encode essential transcription factors or upstream regulatory kinases, which can trigger extensive cascade responses and dramatic expression changes in downstream executor genes through subtle fluctuations in their own expression levels or via post-translational modifications (e.g., phosphorylation), rather than through massive changes in transcript abundance.

Notably, one calcium signal transduction-related gene was detected in each of the two candidate modules: *GH_D06G0540* and *GH_D06G0542*. *GH_D06G0540* encodes a calcium-dependent lipid-binding (CaLB domain) family protein. CaLB proteins act as sensors or effectors of calcium signaling, typically possessing lipid-binding capabilities [39,40]. They localize to the cell membrane or endomembrane system to participate in vesicle trafficking, facilitating the transport of cellulose synthases (CESAs) [41,42] or cell wall very-long-chain fatty acids (VLCFAs) to the cell surface [15,43,44]. Additionally, CaLB proteins may regulate the rearrangement of the microtubule or microfilament cytoskeleton within fiber cells by binding to phospholipids (e.g., phosphatidylinositol), which is crucial for maintaining polarized growth. *GH_D06G0542* functions as a calmodulin-binding receptor-like cytoplasmic kinase (CRCK) gene, serving as a downstream effector in calcium signaling pathways [39,40]. Furthermore, another hub gene related to the regulation of cell wall VLCFAs is *GH_D06G0609*, which is a 3-ketoacyl-CoA synthase (KCS) [41,42] gene that regulates the biosynthesis of VLCFAs, thereby influencing fiber cell elongation and cell wall formation. KCS is also inhibited by *GhBZR3*, which consequently affects fiber development [15,43,44]. In addition to the hub genes which possess distinct functions in regulating fiber development, the DEGs contained within the two candidate modules have also been proven to play vital roles in this process. *GH_D06G0564* encodes an endo-β-1,3-glucanase. Previous studies have indicated that the endo-β-1,3-glucanase *GhPdBG* targets plasmodesmata, which act as intercellular channel switches, to regulate the genetic development of cotton fibers and trichomes [24]. Moreover, the endo-β-1,3-glucanase *GhGLU18* promotes polysaccharide accumulation, cell wall thickening, cellulose synthesis, and the tightening of the fiber spiral pitch, ultimately leading to increased fiber length and strength [25]. *GH_D06G0723* encodes phosphofructokinase 3 (PFK3), a core rate-limiting enzyme in the glycolysis pathway that catalyzes the conversion of fructose-6-phosphate to fructose-1,6-bisphosphate. It determines the amount of glucose allocated for UDP-glucose synthesis, thereby affecting the abundance of cellulose available for cell wall construction [45,46]. *GH_D06G0527* encodes a basic chitinase, which interacts with arabinogalactan proteins (AGPs) or chitin-like oligosaccharides in the cell wall to regulate cell wall relaxation and expansion, participating in cell wall remodeling and elongation [21,47,48]. The identification of these critical genes with explicit functions not only demonstrates the reliability of the strategies and analyses employed in this study but also verifies the importance of the identified candidate modules and genes.

Finally, despite the significant findings in this study, several limitations should be acknowledged. First, regarding our RNA-seq experimental design, RNA from three biological replicates was pooled to construct a single sequencing library in this study. Although this pooling strategy helps to capture the average expression profile and we have excluded abnormal expression data through similar sample testing, it unfortunately eliminates the ability to assess biological variability among individual samples. As a consequence, the statistical robustness of the differentially expressed genes (DEGs) identified in this study is inherently limited. Therefore, the DEG results should be interpreted with caution, and the regulatory roles of the identified candidate genes need to be further validated in future studies using independent biological replicates. Second, for the transcriptome analysis, we utilized five individuals per group representing the extreme phenotypic variations (HH vs. LL). While five biological replicates exceed the minimum statistical requirement for standard RNA-seq and are effective in capturing major trait-related genes, this relatively small sample size might limit the ability to capture the full spectrum of biological variation for highly complex fiber-related traits. Therefore, future studies involving larger natural populations or diverse genetic backgrounds are necessary to further validate the robustness and broad generalizability of the key genes and regulatory modules identified here.

## 4. Materials and Methods

### 4.1. Plant Materials

The F_1_ (BC_6_F_1_) generation was generated by backcrossing CCRI45 (male) to the selected individuals (BC_5_F_2_) harboring the *Seg-D06-2* in Shijiazhuang in the summer of 2023, and self-crossing seeds (BC_6_F_2_) were harvested in Hainan province in the winter of 2023. In 2024, a total of 3324 BC_6_F_2_ plants were developed and fiber collected from individuals in Shijiazhuang experimental farm of the Institute of Cotton, Hebei Academy of Agriculture and Forestry Sciences, with row length of 8 m, row spacing of 0.75 m, and plant spacing of 0.25 m.

The anthesis day of each flower was tagged as 0 days post anthesis (DPA). The developing fibers were sampled from bolls at 0, 5, 10, 15, 20 and 25 DPA; fiber samples were peeled off the ovules and stored in liquid nitrogen for quick-freezing, and then stored at −80 °C until the next step of the experiments.

Naturally opened bolls were collected from the BC_6_F_2_ individuals for phenotype evaluation, including seed cotton weight, fiber weight and fiber strength (FS). LPs were calculated using 100 × (fiber weight)/(seed cotton weight), and the FSs were tested with HFT9000 using HVICC international calibration cotton samples in the Cotton Quality Supervision and Testing Center of the Ministry of Agriculture of China.

Candidate samples with high FS and LP were initially identified based on the top 10% of the phenotypic distribution. By integrating these two traits, five representative samples exhibiting both high FS and high LP (HH) were subsequently selected. Similarly, five representative samples with both low FS and low LP (LL) were selected from the bottom 10% of the phenotypic distribution. Five individual samples were selected for mutual cross-validation for each category (totaling 12 combination types derived from 2 phenotypes across 6 developmental stages) in order to mitigate sampling errors.

### 4.2. RNA Extraction and Sequencing

Total RNA was isolated from the collected fiber of each individual using the Plant RNA Rapid Extraction kit (Molfarming, Nanjing, China). RNA quality and concentration were examined with the Agilent 2100 RNA 6000 Nano kit (Agilent Technologies, Santa Clara, CA, USA). Only RNA samples with OD260/280 = 1.8–2.2, OD260/230 > 2.0 and RNA integrity number (RIN) > 8 were used for RNA sequencing. To minimize sampling batch effects, each biological replicate of all samples was collected within the shortest possible timeframe, and RNA extraction was performed by the same group of operators in a sterile environment. Subsequently, three biological replicates of each sample were pooled in equal molar amounts to construct a single sequencing library. Library construction and sequencing were accomplished by Majorbio Bio-pharm Technology Corporation, Ltd. (Shanghai, China). The mRNA was enriched from total RNA using magnetic beads with Dynabeads™ oligo(dT) (Invitrogen, Carlsbad, CA, USA) for library preparation. A total of 60 libraries was sequenced using the Illumina Novaseq 6000 platform (Illumina, San Diego, CA, USA) with 2 × 150 bp paired-end raw reads.

### 4.3. RNA-Seq Data Analysis

The RNA-seq raw data were processed to filter out the adapter, poly-N, and low-quality reads using Trimmomatic (v0.36) software [49]. The clean data were mapped to the reference genome of TM–1 (*G. hirsutum*) [31] using HISAT2 (v2.2.1) [50]. Gene expression values were estimated using the Subread suite (v1.5.2) [51], and the transcripts per kilo-base of exon model per million mapped reads (TPM) were calculated to measure the gene expression level [52]. Furthermore, Principal Component Analysis (PCA) was employed to cluster and evaluate the samples, facilitating the identification and exclusion of any outlier samples exhibiting anomalous expression data. The differentially expressed genes (DEGs) between samples were identified using the DESeq2 (v1.44.0) R package [53]. The genes with padj ≤ 0.05 and an absolute value of log2 fold change > 2 were defined as significant DEGs. A Venn diagram and volcano plot were drawn using TBtools-II v2.4 [54].

### 4.4. Construction of Gene Co-Expression Networks

Gene co-expression networks were constructed using the R pipeline WGCNA [28]. The genes actively expressed (counts ≥ 10.0) were clustered into co-expression modules, and the correlations between each module eigengene and traits (excellent-FS-LP/low-FS-LP) were used to estimate module–trait associations. The co-expression networks of candidate modules that significantly related to traits were established with an eigengene-based connectivity (KME) value ≥ 0.8 and edge weight value ≥ 0.4, and visualized by Cytoscape v3.5.1 [55]. Furthermore, hub genes, which show the most significant connections in networks, were identified on the basis of their high module membership (KME) values > 0.9 and gene significance > 0.5.

### 4.5. Function Annotation

Gene Ontology (GO) functional enrichment and Kyoto Encyclopedia of Genes and Genomes (KEGG) pathway analyses were performed using TBtools-II v2.4 [54], based on the functional annotation of the TM-1 reference genome [31].

### 4.6. Validation of Key Genes by qRT-PCR

First-strand cDNA was synthesized from the total RNA of the selected sample using the HiScript II Reverse Transcriptase Kit (Vazyme, Nanjing, China). The cDNA was diluted to 100 ng/µL and mixed with TransStart TOP Green qPCR SuperMix (TransGen, Beijing, China) to a total of 20 µL for qRT-PCR. The amplifications were conducted on an ABI Prism 7500 Fast Real-time PCR System (Applied Biosystems, Foster City, CA, USA). Each qRT-PCR reaction included three biological replicates and three technical replicates. The expressions levels were normalized using ACTIN (GenBank: AY305733) as an internal reference and calculated using the 2^−∆∆Ct^ method [56]. The specific primers were designed using Oligo-7 [57] and synthesized by Sangon Biotech (Shanghai, China).

## 5. Conclusions

In conclusion, this study constructed segregating CSSLs targeting the candidate segment *Seg-D06-2* for the simultaneous improvement of FS and LP, and innovatively screened extreme individuals with synchronized improvement for comparative transcriptomic and WGCNA analyses during fiber development. Two reliable co-expression modules significantly associated with the synergistic improvement of FS and LP were uncovered, and seven hub genes along with three DEGs were identified within them. These 10 key genes play pivotal roles in regulating the synergistic development of FS and LP. This study provides valuable genetic resources for the molecular breeding of cotton with simultaneous improvements in FS and LP and offers valuable insights for further elucidating the molecular mechanisms underlying their synergistic development.

## Figures and Tables

**Table 1:** Phenotypes of fiber strength (FS) and lint percentage (LP) for extreme samples.

Phenotypic Type	Sample ID	FS (cN/tex)	LP (%)
High FS & High LP (HH)	HS1	34.30	38.57
	HS2	34.26	38.60
	HS3	34.16	38.56
	HS4	34.10	38.51
	HS5	33.45	39.20
Low FS & Low LP (LL)	LS1	24.69	26.73
	LS2	25.11	26.74
	LS3	24.70	26.80
	LS4	24.65	27.09
	LS5	25.12	26.21

## Data Availability

All raw sequencing data generated in this study have been deposited in the Genome Sequence Archive (GSA) in the National Genomics Data Center (CNCB-NGDC), China National Center for Bioinformation (https://ngdc.cncb.ac.cn/gsa) (accessed on 5 June 2019), under BioProject accession number PRJCA063304 with submission ID subCRA069468. These data are publicly accessible.

## Supplementary Materials

The following supporting information can be downloaded at: https://www.mdpi.com/article/10.3390/ijms27104180/s1.
